# Case report: Successful management of *Parvimonas micra* pneumonia mimicking hematogenous *Staphylococcus aureus* pneumonia

**DOI:** 10.3389/fmed.2022.1017074

**Published:** 2022-10-28

**Authors:** Yanmei Feng, Chunxia Wu, Xiaohui Huang, Xia Huang, Li Peng, Rui Guo

**Affiliations:** ^1^Department of Respiratory and Critical Care Medicine, The First Affiliated Hospital of Chongqing Medical University, Chongqing, China; ^2^Department of Respiratory and Critical Care Medicine, Liangping People’s Hospital, Chongqing, China; ^3^Department of Critical Care Medicine, Chongqing University Three Gorges Hospital, Chongqing, China; ^4^Department of Critical Care Medicine, The First Affiliated Hospital of Chongqing Medical University, Chongqing, China

**Keywords:** *P. micra* pneumonia, dysbiosis, oral flora, poor oral hygiene, new etiology

## Abstract

*Parvimonas micra* is an anaerobic Gram-positive coccus frequently found in the oral cavity and gastrointestinal tract, but rarely in the lung. Therefore, pneumonia caused by *P. micra* is also rare. Although there are some reports of *P. micra* related pneumonia due to aspiration or blood-borne infection with definite remote infection source, there are no reported cases of hematogenous *P. micra* pneumonia in healthy adults lacking a remote source of infection. Herein, we described the intact disease of *P. micra*-related pneumonia mimicking hematogenous *Staphylococcus aureus* pneumonia in terms of chest imagery and diagnosed *via* metagenomic next-generation sequencing (mNGS). Interestingly, there was no clear remote pathogenic source identified in the patient. Microbiome analysis revealed dysbiosis of the oral flora possibly related to poor oral hygiene and a long history of smoking. The patient was treated with moxifloxacin for 3 months. Ultimately, computed tomography (CT) of the chest showed total resolution of the lung lesion. Clinicians need to update the etiology of community-acquired pneumonia. When antibiotic therapy is not effective, pathogen examination becomes very important. New methods of pathogen detection such as mNGS should be employed to this end. For the treatment of *P. micra* pneumonia, no standardized course of treatment was reported. Imaging absorption of lung infections may provide a more objective guidance for the duration of antibiotics in *P. micra* pneumonia.

## Introduction

*Parvimonas micra*, previously known as *Peptostreptococcus micros* and *Micromonas micros*, is commonly found on human skin, in oral cavity, and among gastrointestinal flora (1). It is an anaerobic Gram-positive coccus with a diameter of 0.3–0.7 μm. This opportunistic pathogen is frequently isolated from infected root canals of teeth with chronic apical periodontitis, whereas remote infections are rare (2). A few sporadic cases of remote infection in patients with underlying diseases or a recent oral operation have been reported (3–6).

Because it is anaerobic, *P. micra* is not a dominant bacterium in aerobic environments, including the lung. Although there are some reports of *P. micra* pneumonia due to aspiration, hematogenous *P. micra* pneumonia is rare (7–9). Moreover, to the best of our knowledge, there are no reports of *P. micra*-related pneumonia induced by bloodstream infection without a definitive, remote pathogenic source.

The gold standard method of diagnosing *P. micra* infection is microbiological examination. However, it is difficult to detect using traditional cultural methods (10). Recently developed non-culture methods, such as metagenomic next-generation sequencing (mNGS), provide an alternative for the identification of unknown pathogens (11). As a new tool, mNGS is any of several high-throughput sequencing methods whereby billions of nucleic acid fragments can be simultaneously and independently sequenced. It can be used to precisely and rapidly identify potential pathogens regardless of pathogen type (bacterium, virus, fungus, parasite, and so on) (12). The mNGS is a promising method for diagnosing sophisticated infections, especially severe pneumonias require stay in the intensive care units (13, 14).

Herein, we describe *P. micra* pneumonia mimicking hematogenous *Staphylococcus aureus* pneumonia in terms of chest imagery and diagnosed *via* mNGS in pleural effusion. Interestingly, there was no definitive, remote pathogenic source identified in the patient. A cautious approach to diagnosis is necessary for rare cases such as this to avoid misdiagnosis, and clinical knowledge of *P. micra*-related pneumonia should be updated.

## Case presentation

**FIGURE 1:**
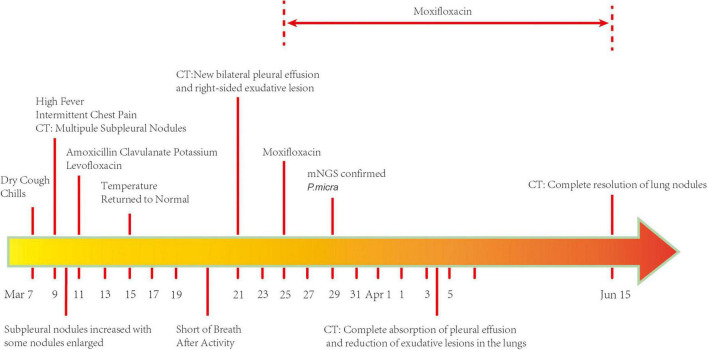
Timeline of the clinical course and duration of treatment.

**FIGURE 2:**
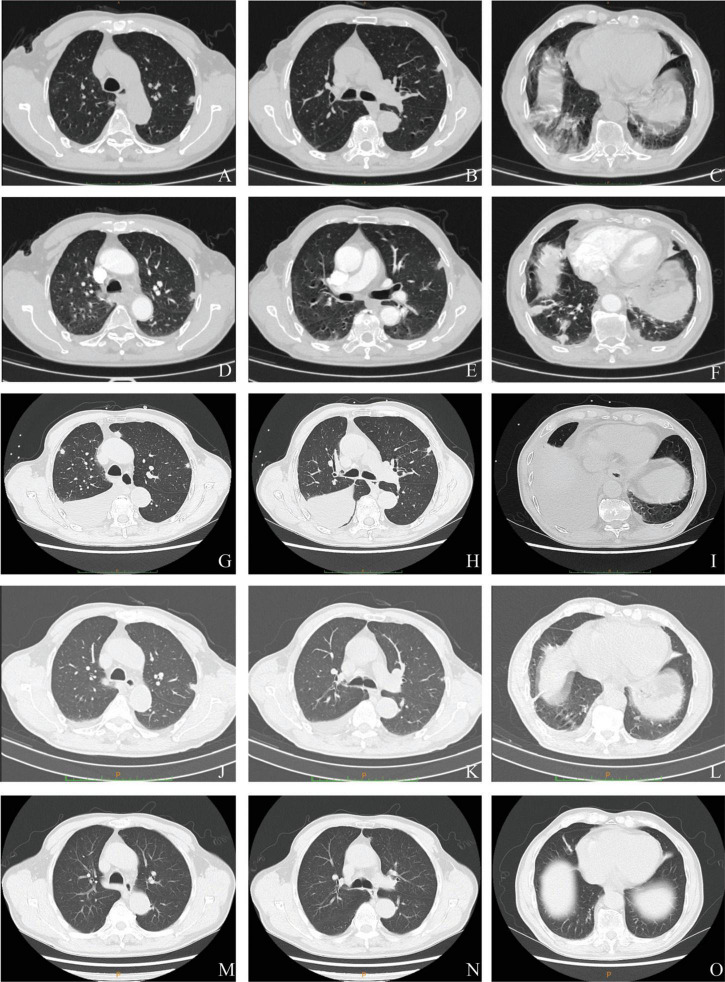
Serial chest computed tomography (CT) scans of the patient with *Parvimonas micra* pneumonia. **(A–C)** The initial CT scan on 9 March 2022 (2 days after symptom onset) shows subpleural nodules in both lungs, some nodules with ground glass appearance, and a few exudative inflammatory lesions in both lower lungs. **(D–F)** A CT on the next day (10 March 2022) showed more nodules and exudative lesions in both lungs and some enlarged nodules. **(G–I)** A follow-up scan (21 March 2022) after treatment with amoxicillin clavulanate potassium and levofloxacin showed multiple subpleural nodules with newly emerged bilateral pleural effusion and an exudative lesion in the right lung. **(J–L)** Another CT (4 April 2022) showed complete absorption of the pleural effusion and a reduced exudative lesion. **(M–O)** Another CT after moxifloxacin treatment for 3 months (15 June 2022) exhibited an almost normal chest image.

A 74-year-old man was admitted to the Department of Respiratory and Critical Care Medicine of the First Affiliated Hospital of Chongqing Medical University on 25 March 2022 with a complaint of dry cough, fever, and intermittent chest pain for 17 days and shortness of breath after activity for 4 days. He denied night sweats and weight loss. There was no history of hemoptysis, recent contact with tuberculosis or irritant gas, or any other underlying health conditions, except for 20-year smoking history. When the symptoms first occurred, the patient underwent a series of computed tomography (CT) scans. At that time, he was treated with amoxicillin clavulanate potassium for 4 days and with levofloxacin for 6 days (Figure 1). His chest pain was significantly relieved and his temperature returned to normal. However, the patient continued to cough intermittently and experience shortness of breath after activity. As shown in Figure 2, consecutive CT images showed an increasing number of nodules and exudative inflammatory lesions with a newly emerged pleural effusion. Physical examination indicated normal vital signs (Body temperature: 36.2°C, Pulse rate: 70/min, Respiratory rate: 20/min, Blood pressure: 146/74 mmHg) but asymmetric breath sounds without rales or wheezing. Blood gas analysis indicated a Pondus Hydrogenii (PH) of 7.48, a Partial Pressure of Carbon Dioxide (PCO_2_) of 37 mmHg, Oxygen Permeance (PO_2_) of 73 mmHg, Bicarbonate (HCO_3_^–^) of 27.6 mmol/L, and oxygen saturation (SO_2_) of 96%. His leukocyte levels were normal, but his erythrocyte sedimentation rate was elevated, at 61 mm/h.

According to his clinical symptoms and the dynamic changes observed in chest CT, we highly suspected an infectious lung disease. Because of the non-specific clinical manifestations, pathogenetic examination was very important for this patient. Therefore, we collected microbiological samples and performed routine bacteria culture. The GeneXpert MTB/RIF assay and mNGS were performed on 29 March 2022, 4 days after admission, in an attempt to identify pathogenic bacteria. Meanwhile, his antibiotic treatment was switched to moxifloxacin to better fight against Gram-positive bacteria and covered the antibacterial spectrum of anaerobic bacteria. As shown in Table 1, the laboratory tests, such as Galactomannan Antigen Testing (GM), β-D-glucan fungal antigen test (G test), and tests for cryptococcal pod antigen, were negative; however, the patient was positive for mycoplasma antibody (IgM). The pleural effusion was exudate consisting of 44% leukocyte. Samples of it were sent for routine bacteria culture and were analyzed using the GeneXpert MTB/RIF assay and mNGS. BALF samples were also subjected to routine bacteria culture and GeneXpert MTB/RIF assay. All samples returned negative in all of the tests except mNGS, which identified four specific reads of *P. micra* with 9% relative abundance from pleural effusion samples.

**TABLE 1:** Laboratory tests for clinical pathogen identification.

	Normal range	Result
**Blood**		
Total lymphocyte count	1,752–2,708/μL	1,159/μL
CD4 + T	561–1,137/μL	460/μL
CD8 + T	464–754/μL	368/μL
B lymphocyte	180–324/μL	156/μL
Native killer cells	175–567/μL	116/μL
CD4 + /CD8 +	0.89–2.01	1.25
Tuberculosis antibody	Negative	Negative
GM test	<0.5	0.09
Cryptococcal capsular antigen	Negative	Negative
Antinuclear antibody spectrum	Negative	Negative
ANCA	Negative	Negative
Anti-*legionella pneumophila* serum type I antibody IgM	Negative	Negative
Anti-*chlamydia pneumoniae* antibody IgM	Negative	Negative
Anti-*rickettsia Q fever* IgM antibody	Negative	Negative
Anti-*chlamydia pneumoniae* antibody IgM	Negative	Negative
Anti-*adenovirus* antibody IgM	Negative	Negative
Anti-*respiratory syncytial virus* antibody IgM	negative	Negative
IgM antibody against *influenza A virus*	Negative	Negative
Anti-*influenza B virus* IgM antibody	Negative	Negative
*Mycoplasma pneumoniae* antibody IgM	Negative	Positive
**BALF**		
Bacterial smear	Negative	Negative
Fungal smear	Negative	Negative
Bacterial culture	Negative	Negative
Fungal culture	Negative	Negative
Smear of *Mycobacterium tuberculosis*	Negative	Negative
Tubercle bacillus culture	Negative	Negative
**Pleural Effusion**		
Number of nucleated cells	(0–8) × 10^6^/L	3,677 × 10^6^/L
Ratio of multinucleated cells		44%
Ratio of monocytes		56%
Smear of *Mycobacterium tuberculosis*	Negative	Negative
Culture of pleural effusion	Negative	Negative
GeneXpert MTB/RIF assay	Negative	Negative
Tubercle bacillus culture	Negative	Negative
mNGS		*P. micra* (4 copies with 9% relative abundance)

**FIGURE 3:**
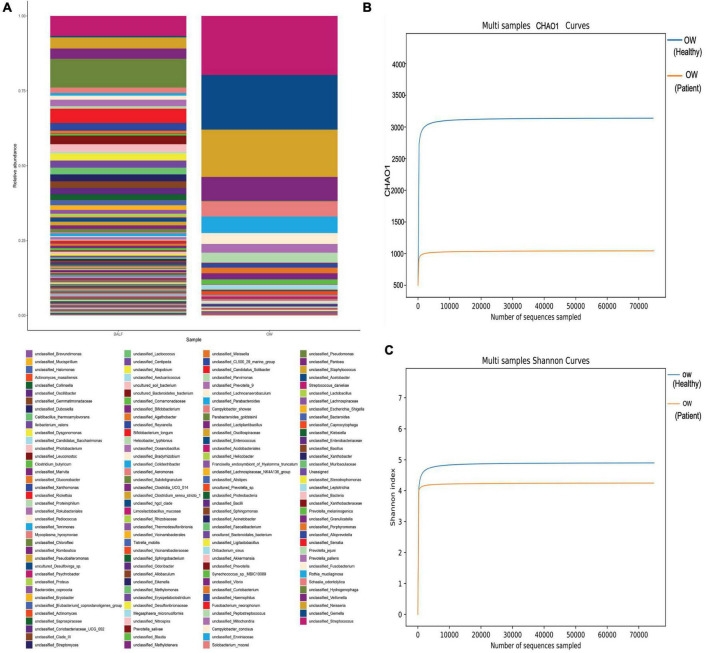
Comparison of the oral and lung microbiome (heatmap). **(A)** No significant differences were found in *P. micra* between oral wash and BALF samples (0.11% in BALF *vs*. 0.13% in oral wash). **(B,C)** The α diversity of the oral microbiome comparing the patient to a healthy man. Both the Chao1 and Shannon indexes were lower in the patient (Chao1: 986 *vs*. 3,079, Shannon index: 4.244 *vs*. 4.829).

To search for the source of infection, enhanced CT of the oropharyngeal and abdomen as well as cardiac ultrasound were conducted. A medical history inquiring about toothache, periodontitis, and oral invasive operations was conducted, and the patient denied underlying oral diseases. No abscesses were detected in any CT image, and cardiac ultrasound revealed a lack of bacterial emboli. However, an examination of the oral cavity indicated that most tooth crowns of this patient were missing, while the roots remained. Oropharyngeal CT also suggested multiple root remnants with partial alveolar bone resorption. Further inquiries revealed that the patient wore dentures for an extended period of time; these were cleaned and soaked in normal tap water that was not changed every day. Based on the poor oral hygiene and colonization of the oral cavity with *P. micra*, we intended to conduct mNGS to analyze oral and lung pathogens. However, because of the high cost of mNGS, we had to analyze the oral and lung microbiome by 16s rRNA gene sequencing. Interestingly, the results identified *Parvimonas* not only in the oral cavity but also in the BALF (Figure 3). Compared to the oral flora of healthy men (15), the patient’s α diversity of the oral cavity was significantly reduced (Figure 3). Based on these factors, we speculated that the oral cavity could have been the potential infection source. Moxifloxacin treatment was continued. Fortunately, a follow-up chest CT on 4 April 2022 showed complete absorption of pleural fluid and significant resolution of the exudative lesions (Figure 2). Given that the adjusted treatment was effective, moxifloxacin was continued for 3 more months. Finally, a chest CT on 15 June 2022 showed almost total resolution of the lung lesion (Figure 2).

## Discussion

*Parvimonas micra* is part of the normal microbiome of the oral cavity, gastrointestinal tract, and skin. However, it can be a pathogen in patients with impaired immune function or with underlying disease. Cases of *P. micra* infection of the hip joint, pericardium, cervix, liver, and brain have been reported (4, 16–18). However, because of the aerobic environment of the lung, this pathogen is not common in the lung and it rarely causes pneumonia. Tsyshi et al. (19) reported a case of hematogenous lung abscess caused by *P. micra* in an 85-year-old male with diabetes. In that case, remote sites of infection were detected: apical periodontitis and an infratemporal fossa abscess.

Here, we report a case of *P. micra* pneumonia mimicking hematogenous *Staphylococcus aureus* pneumonia in terms of chest imagery without a definitive remote site of infection. There was no direct evidence of blood-borne infection, but we still highly suspected hematogenous infection based on the chest imagery. According to microbiome analyses of the oral cavity and lung by 16s rRNA gene sequencing, the patient’s poor oral hygiene was suspected to be related to the infection. However, as mentioned above, *P. micra* is a normal component of the oral and intestinal flora in healthy people. The patient in our case denied any underlying disease, except for a long history of heavy smoking. It has been reported that smoking tobacco disrupts immune homeostasis, resulting in a variety of illnesses (20). It also affects oral microbiota composition (21). The level of *P. micra* and other bacteria in the oral cavity is higher in smokers than in non-smokers (8). We also noted poor hygiene with suspected periodontitis and decreased α diversity in the oral microbiome of our patient, accompanied by similar copies of *P. micra* between oral wash and BALF samples. Therefore, we speculate that our patient’s *P. micra* pneumonia may have been induced by dysbiosis of his oral microflora and tobacco smoking.

Common hematogenous lung abscesses always present with high fever, cough with or without sputum, and multiple small subpleural nodules with dynamic change in CT scans. According to a previous systematic review (22), the common pathogens of hematogenous lung abscesses are methicillin-sensitive *Staphylococcus aureus* (MSSA) and methicillin-resistant *Staphylococcus aureus* (MRSA). Isolation of *P. micra* as the causative pathogen of a hematogenous lung abscess is rare. Little is known about the clinical characteristics of *P. micra* hematogenous lung infections. In our case, the patient showed the much similar manifestations to hematogenous lung infection. Because there are no known specific symptoms of hematogenous *P. micra* pneumonia, it was extremely critical to conduct a microbiological examination. Unfortunately, however, it is difficult to detect this microbe using conventional culture methods. Hence, we used the mNGS technique, which has a wide detection range and does not need to specify the suspected causative microorganism in advance. In addition, comparing to conventional culture methods, the results are obtained very quickly (2–3 days *vs*. 5–7 days for conventional methods) and the positive rate of identified pathogens is much higher (95 *vs*. 54%) (23).

*Mycoplasma pneumoniae* IgM is an early antibody that appears after infection, and it usually appears only after 4–5 days and lasts 1–3 months or more. A positive IgM for *Mycoplasma pneumoniae* may indicate current infection with *Mycoplasma pneumoniae* or it may not, as some patients may show prolonged IgM seropositivity caused by prior *Mycoplasma pneumoniae* infection (24). So, in our case, the patient was thought to be a false positive *Mycoplasma pneumoniae* IgM and diagnosed with *P. micra* detection in pleural effusion with mNGS in our case. Normal pleural effusion fluid is sterile, and it can be identified as pathogenic if bacteria are detected in it. Therefore, we confirmed that *P. micra* was the pathogen causing the pulmonary infection in our patient rather than *Mycoplasma pneumoniae*.

Although *P. micra* is typically susceptible to antibiotics such as penicillin, clindamycin, metronidazole, and imipenem, drug-resistant strains may also exist (9, 25). In this case, amoxicillin clavulanate potassium and levofloxacin were administered successively. Unfortunately, the clinical symptoms of the patient remained poorly controlled and the lesions in the lung became exacerbated. So we highly suspected that the patient suffered from drug-resistant strains, even *P. micra* was theoretical sensitivity to penicillin (26). Hence, if possible, we recommend acquiring drug sensitivity test along with the pathogenic cultures or drug-resistant gene test, which may provide us with the necessary information to develop precise treatments.

The previous studies have suggested that the durations of antibiotic therapy for *P. micra* pneumonia are highly variable (8) and there is little evidence of hematogenous *P. micra* pneumonia. Watanabe et al. (7) reported a case of *P. micra*-related hematogenous lung abscess in which chest CT showed that the lung lesions resolved after 1 month of antibiotic treatment with ampicillin sulbactam. Ubukata et al. (19) reported a similar case where antibiotics were continuously administered for 3 months. Similarly, we administered moxifloxacin for 3 months in our patient. Considering the large variation in antibiotic duration to treat *P. micra* pneumonia, we recommend using lung imaging instead to guide the antibiotic regimen.

There were some limitations to this study. Due to the usage of antibiotics prior to admission and the relatively high cost of mNGS in the clinical setting, blood samples were not used for mNGS tests. In addition, the patient’s oral and pulmonary flora were not analyzed after treatment, and thus any differences pre- and post-treatment with moxifloxacin could not be determined.

## Conclusion

In summary, we report a case of *P. micra* pneumonia in a patient without any apparent underlying diseases and no remote site of infection. Clinicians need to update the etiology of community-acquired pneumonia, especially in smoking patients with poor oral hygiene. When antibiotic therapy is ineffective, early initiation of mNGS is critical for the rapid screening of rare pathogens such as *P. micra*. If possible, we recommend choosing a reasonable antibiotic based on drug sensitivity tests of *P. micra* to avoid possible drug resistance. Finally, lung infection absorption as determined *via* imaging may provide more objective guidance of treatment than the duration of antibiotic treatment.

## Ethics statement

Written informed consent was obtained from the individual(s) for the publication of any potentially identifiable images or data included in this article.

## Author contributions

CW, XHH, and XH were involved in the patient’s clinical treatment. YF and LP contributed to the diagnosis. RG analyzed the pathological and CT images. YF integrated all information and wrote the manuscript. LP and RG provided critical guidance and revisions for YF throughout the writing process. All authors contributed to the article and approved the submitted version.
